# Wave Packet Approach to Adiabatic and Nonadiabatic Dynamics of Cold Inelastic Scatterings

**DOI:** 10.3390/molecules27092912

**Published:** 2022-05-03

**Authors:** Bayaer Buren, Maodu Chen

**Affiliations:** Key Laboratory of Materials Modification by Laser, Electron, and Ion Beams (Ministry of Education), School of Physics, Dalian University of Technology, Dalian 116024, China; burin@mail.dlut.edu.cn

**Keywords:** quantum wave packet method, inelastic scattering, cold collisions

## Abstract

Due to the extremely large de Broglie wavelength of cold molecules, cold inelastic scattering is always characterized by the time-independent close-coupling (TICC) method. However, the TICC method is difficult to apply to collisions of large molecular systems. Here, we present a new strategy for characterizing cold inelastic scattering using wave packet (WP) method. In order to deal with the long de Broglie wavelength of cold molecules, the total wave function is divided into interaction, asymptotic and long-range regions (IALR). The three regions use different numbers of ro-vibrational basis functions, especially the long-range region, which uses only one function corresponding to the initial ro-vibrational state. Thus, a very large grid range can be used to characterize long de Broglie wavelengths in scattering coordinates. Due to its better numerical scaling law, the IALR-WP method has great potential in studying the inelastic scatterings of larger collision systems at cold and ultracold regimes.

## 1. Introduction

The study of molecular collisions at low temperatures has attracted great interest due to the special properties of cold molecules [1,2,3,4,5]. Cold atoms and molecules can be prepared in a single quantum state, and their motion is controlled by quantum effects. Cold atoms and molecules have extremely long de Broglie wavelengths and the long-range potentials play an important role in collisions. In addition, only one or a few partial waves contribute to collisions, allowing for resonance in cross sections to have better resolution. These properties of cold molecules are of great significance for understanding the nature of molecular collisions, and thus promote the study of molecular collisions at cold and ultracold temperatures [6,7,8,9].

Molecular inelastic collisions are common in the interstellar medium [10], combustion [11] and chemical lasers [12]. The study of energy transfer process in molecular inelastic collisions is very important for understanding many physical phenomena. The distribution of intramolecular states in molecular inelastic scattering is affected by many factors at low temperature, such as long-range van der Waals interaction, shape resonance, Feschbach resonance, initial orientation of molecules, etc. Additionally, only the full quantum dynamics calculation can reveal the influence of these factors on the molecular inelastic scattering. Up to now, most of the theoretical studies on inelastic scattering of cold/ultracold molecules have used the time-independent close coupling (TICC) method [13,14,15,16]. For TICC method, the numerical costs scale as *N* [3], where *N* is the number of basis set used in calculations. Therefore, TICC method is currently only applied to collision systems with fewer open channels. At present, full-dimensional TICC calculations can be performed for most of the inelastic collisions of triatomic systems and some tetratomic systems. In order to improve the practicability of TICC method, many approximations have been proposed to reduce the number of channels in TICC calculation. The most widely used approximation is the rigid rotor model [17], which ignores the vibrational dimension of the colliding molecules and greatly reduces the number of channels. However, the rigid rotor model will produce large errors when there are strong interactions between the colliding molecules [18]. For collision with *J* > 0, where *J* is total angular momentum quantum number, the Coriolis coupling (CC) also greatly increases the number of channels in TICC calculations. In order to reduce the number of channels caused by CC coupling, many approximation methods have been proposed, such as minimum-*K* scheme approximation [19], infinite order sudden approximation [20], coupled-states approximation or centrifugal sudden approximation [21] and recently developed coupled-states approximation including the nearest neighbor Coriolis coupling [22]. Despite these approximations, TICC is still difficult to extend to complex collision systems.

The wave packet (WP) method is another quantum dynamic method commonly used to describe molecular collisions, and its numerical costs scale as NlogN. Compared with the TICC method, WP method has better scalability, so it is widely used in the study of quantum dynamics of more complex systems. [23,24,25,26,27] However, the WP approach faces some challenges in the study of molecular collisions at cold and ultracold regimes. On the one hand, due to the extremely long de Broglie wavelengths of cold molecules, a large number of grids need to be used in the scattering dimension, which greatly increases the computer memory and computing time required by the WP calculations. On the other hand, wave packet moves very slowly at low temperatures, and the required propagation time increases rapidly as the collision energy decreases. In order to extend the WP calculation to molecular collisions at low temperatures, the grid method used in the calculation needs to be improved. Zhang et al. [28] proposed the L-shaped grid method, which divided the total grid space into interaction region and asymptotic region with different number of vibrational basis, thus saving a large number of grids. In a previous work, we further improved the L-shaped grid method. Zhao et al. [29] proposed the interaction-asymptotic region decomposition (IARD) grid method, in which the hyperspherical and Jacobi coordinates are used for interaction and asymptotic regions, respectively. Both IARD and improved L-shaped grid method have been successfully applied to the study of molecular collision dynamics at low temperatures. However, when the collision temperature decreases to cold or even ultracold, the number of grids required by the asymptotic region is much larger than that required by the interaction region, which makes the calculation of improved L-shaped method and IARD method very difficult. To this end, Huang et al. [30] proposed the interaction-asymptotic-long-range region (IALR) method and successfully extended the WP calculation to ultracold regimes.

So far, the IALR-WP method has been successfully used to study atom–diatom [31,32] and diatom–diatom [33] reactions at cold and ultracold temperatures, but has not been applied to the study of inelastic scattering dynamics. Since the WP method can be extended to more complex systems, it is of great significance to study inelastic scattering at cold and ultracold temperatures by using IALR-WP method. In this work, we further extend the IALR-WP method to treat molecular inelastic scattering at cold temperatures, and demonstrate its accuracy by applying it to several typical collision systems.

## 2. Theory

**Figure 1:**
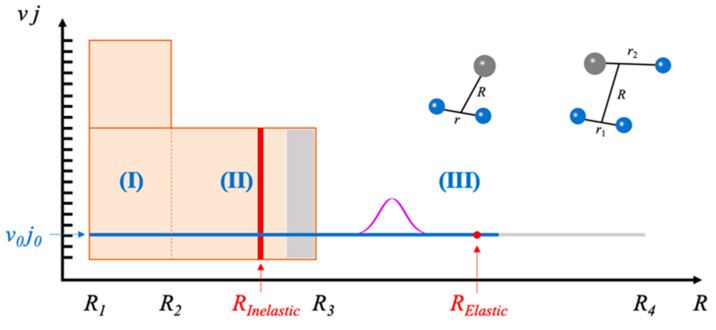
Illustrative drawing of the grids used for the IALR-WP method. Red curves represent the projection plane where time-independent wave functions are collected, gray represents the region of damping functions.

In this section, the IALR-WP method for solving bimolecular inelastic scattering is introduced. In the IALR-WP method, total grid space is decomposed into interaction region (*R* ∈ [*R*_1_, *R*_2_]), asymptotic region (*R* ∈ [*R*_2_, *R*_3_]) and long-range region (*R* ∈ [*R*_3_, *R*_4_]) by the radial distance between colliding particles, as shown in Figure 1.

The total time dependent wave packet in body-fixed (BF) frame can be expanded as

(1) $$\Psi^{JMp}(R,r,t)=\sum_{n,v,j,K}F_{nvjK}^{JMp}(t)u_{n}^{vj}(R)\psi_{vj}$$

where ***R*** is radial distance between colliding atoms/molecules, and ***r*** represents internal degree of molecule. *u_n_^vj^*(*R*) is radial basis function, and *ψ_vj_* is ro-vibrational wave function of molecule. For A + BC collisions, *v* and *j* are vibrational and rotational quantum states of BC molecule, respectively. For AB + CD collisions, *v* denotes (*v*_1_, *v*_2_) and *j* denotes (*j*_1_, *j*_2_), where *v*_1_ (*v*_2_) and *j*_1_ (*j*_2_) are vibrational and rotational quantum states of AB (CD) molecule, respectively.

In the IALR-WP method, different numbers of *ψ_vj_* function are used for three regions. Three sets of wave functions Ψ^I^, Ψ^II^ and Ψ^III^ are defined in the calculation, corresponding to the interaction, asymptotic and long-range regions, respectively. The long-range region is mainly used to describe the initial wave packet and the elastic scattered wave packet, so the Ψ^III^ is defined in the range of *R* ∈ [*R*_1_, *R*_4_], and only a single *ψ_vj_* with *v* = *v*_0_, *j* = *j*_0_ is included. The asymptotic region is mainly used to describe the inelastic scatterings, so the Ψ^II^ is defined in the range of *R* ∈ [*R*_1_, *R*_3_], and a few *ψ_vj_* functions with *v* ∈ [0, *v*_Asy_] and *j* ∈ [0, *j*_Asy_] are included. The interaction region is mainly used to describe the reactive scatterings, so the Ψ^I^ is defined in the range of *R* ∈ [*R*_1_, *R*_2_], and a large amount of *ψ_vj_* functions with *v* ∈ [0, *v*_Int_] and *j* ∈ [0, *j*_Int_] are included. It can be found that there are overlapping regions between the three wave functions. During wave packet propagation, three wave functions exchange information with each other through the overlapping regions. The ro-vibrational state dependent radial basis function is defined as

(2) $$u_{n}^{vj}(R)=\{\begin{array}{l} \sqrt{C^{I}}\sin(n\pi C^{I}R)\;,\;for\;\Psi^{I}\;\\ \sqrt{C^{II}}\sin(n\pi C^{II}R)\;,\;for\;\Psi^{II}\;\\ \sqrt{C^{III}}\sin(n\pi C^{III}R),\;for\;\Psi^{III}\; \end{array}$$

with

(3) $$C^{X}=\frac{1}{(N_{R}^{X}+1)\Delta R},\;(X=I,II,III)$$

where *N_R_*^I^, *N_R_*^II^ and *N_R_*^III^ are grid numbers used in Ψ^I^, Ψ^II^ and Ψ^III^, respectively. Δ*R* is the step size.

The initial wave packet is product of Gaussian wave packet *G*(*R*) and initial ro-vibrational state wave function. The *G*(*R*) is given as

(4) $$G(R)={\left(\frac{2}{\pi \delta^{2}}\right)}^{1/4}\exp\left[-\frac{{\left(R-R_{0}\right)}^{2}}{\delta^{2}}-i\sqrt{2\mu_{R}E_{0}}R\right]$$

where, *μ_R_* is the reduced mass for *R* coordinate. *R*_0_ and *δ* define the position and width of the initial wave packet, respectively, and *E*_0_ is the mean collision energy.

The initial wave packet is propagated by second-order split-operator method [34], which is described by

(5) $$\Psi (t+\Delta_{t})=\exp(-i\hat{V}\Delta_{t}/2)\exp(-i\hat{T}\Delta_{t})\exp(-i\hat{V}\Delta_{t}/2)\Psi (t)$$

where $\hat{T}$ and $\hat{V}$ are the kinetic energy operator and reference potential energy. For non-adiabatic calculations, the potential energy operator is not diagonal in the diabatic representation, the wave packet needs to be first transformed to the adiabatic representation when operating the potential energy terms:

(6) $$\begin{array}{ll} \exp(-i\hat{V}\Delta_{t}/2) & =\exp(-i\left[\begin{array}{cc} V_{11} & V_{12}\\ V_{21} & V_{22} \end{array}\right]\Delta_{t}/2)\\ & =T^{†}\left[\begin{array}{cc} \exp(-iE_{1}\Delta_{t}/2) & 0\\ 0 & \exp(-iE_{2}\Delta_{t}/2) \end{array}\right]T, \end{array}$$

where *E_i_* and **T** are eigenvalues and eigenvectors of the reference diabatic potential energy matrix.

In the IALR-WP method, different damping functions are used for asymptotic and long-range regions. The ro-vibrational state dependent damping function for the Ψ^II^ has following form:

(7) $$D_{vj}^{II}(R)=\{\begin{array}{l} \exp\left[-\Delta_{t}C_{R}^{II}{\left(\frac{R-R_{a}^{II}}{R_{b}^{II}-R_{a}^{II}}\right)}^{2}\right]\;if\;v\neq v_{0},\;j\neq j_{0},\;R>R_{a}^{II}\\ 1\;elsewhere \end{array}$$

and the damping function for the Ψ^III^ has following form:

(8) $$D_{}^{III}(R)=\{\begin{array}{l} \exp\left[-\Delta_{t}C_{R}^{III}{\left(\frac{R-R_{a}^{III}}{R_{b}^{III}-R_{a}^{III}}\right)}^{2}\right]\;if\;R>R_{a}^{III}\\ 1\;elsewhere \end{array}$$

where, *C_R_*^II^ (*C_R_*^III^) defines the strength of damping function, with *R_a_*^II^ (*R_a_*^III^) and *R_b_*^II^ (*R_b_*^III^) as the corresponding starting and ending points of damping functions. Since reactive scattering is not involved in this work, the damping function is not used for *r* coordinate in the calculations.

To collect the elastic and inelastic scattering information form WP calculations, a projection plane is set in the asymptotic region. After the propagation of wave packet, the time-independent scattering wavefunction at projection plane *ψ*^+^(*E*;*R*_∞_) can be obtained by a time-energy Fourier transform of time-dependent wave packet at the projection plane. The state-to-state scattering S-matrix is obtained by

(9) $$S_{f\leftarrow i}^{}(E)=\frac{1}{\alpha (E)}\sqrt{\frac{k_{f}}{2\pi \hbar^{2}\mu_{R}}}h_{l_{f}}(k_{f}R_{\infty })\langle \chi_{v_{f}j_{f}}|\psi^{+}(E;R_{\infty })\rangle$$

where *k_f_* is the momenta in the exit channel, $h_{l_{f}}$ is outgoing Ricatti–Hankel function, $\chi_{v_{f}j_{f}}$ is molecular ro-vibrational wavefunction. The *R*_∞_ is position of the projection plane. The *α*(*E*) is amplitude of the initial Gaussian wave packet at a specified collision energy, and given by

(10) $$\alpha (E)=\sqrt{\frac{\mu_{R}}{2\pi \hbar^{2}k_{v_{0}j_{0}}}}\int h_{l_{0}}(k_{v_{0}j_{0}}R)G(R)dR$$

where, $k_{v_{0}j_{0}}$ is the momenta in the entrance channel, *l*_0_ is the initial orbital angular momentum quantum number.

Normally, in the well-established WP method, the projection plane should be set between the tail of the initial wave packet and the starting point of the damping function. However, in the IALR-WP method, the initial wave packet is always constructed in the long-range region, behind the damping function in the asymptotic region. This means that we cannot use one projection plane to collect both elastic and inelastic information. Since elastic and inelastic scattered waves are treated separately in the IALR-WP method, it allows us to use two different projection planes to collect elastic and inelastic information separately. One projection plane is placed in the asymptotic region to collect information of inelastic scattering, and the other is placed in the long-range region to collect information of elastic scattering, as shown in Figure 1. Since the calculation of inelastic scattering wavefunction is independent from elastic one, and the inelastic scattering projection plane is just set at the asymptotic region, the needed total propagation time is greatly shortened for inelastic scattering compared with elastic scattering.

After propagation for a series of *J* and *l*_0_, the state-to-state inelastic cross section for atom–diatom collision is obtained by

(11) $$\sigma_{v^{\prime }j^{\prime }\leftarrow vj}(E)=\frac{\pi }{(2j+1)k_{vj}^{2}}\sum_{J}\sum_{KK^{\prime }}(2J+1){\left|S_{v^{\prime }j^{\prime }K^{\prime }\leftarrow vjK}^{J}(E)\right|}^{2}$$

Additionally, diatom–diatom inelastic scattering cross section is obtained by

(12) $$\begin{array}{ll} \sigma_{{v^{\prime }}_{1}{j^{\prime }}_{1}{v^{\prime }}_{2}{j^{\prime }}_{2}\leftarrow v_{1}j_{1}v_{2}j_{2}}(E)= & \frac{\pi }{(2j_{1}+1)(2j_{2}+1)k_{v_{1}j_{1}v_{2}j_{2}}^{2}}\\ & \sum_{J}\sum_{KK^{\prime }}\sum_{j_{12}{j^{\prime }}_{12}}(2J+1){\left|S_{{v^{\prime }}_{1}{j^{\prime }}_{1}{v^{\prime }}_{2}{j^{\prime }}_{2}\leftarrow v_{1}j_{1}v_{2}j_{2}}^{J}(E)\right|}^{2} \end{array}$$

## 3. Results and Discussion

In this section, in order to test the accuracy of the IALR-WP method in characterizing inelastic scattering at cold temperatures, the dynamics calculations are performed for three typical collision systems. The three collision systems selected in this paper represent three different inelastic collision cases: the adiabatic atom–diatom collision, the non-adiabatic atom–diatom collision and the adiabatic diatom–diatom collision. The adiabatic calculations results are compared with those of the TICC method, and the non-adiabatic calculations results are compared with those of the L-shaped WP method.

**Figure 2:**
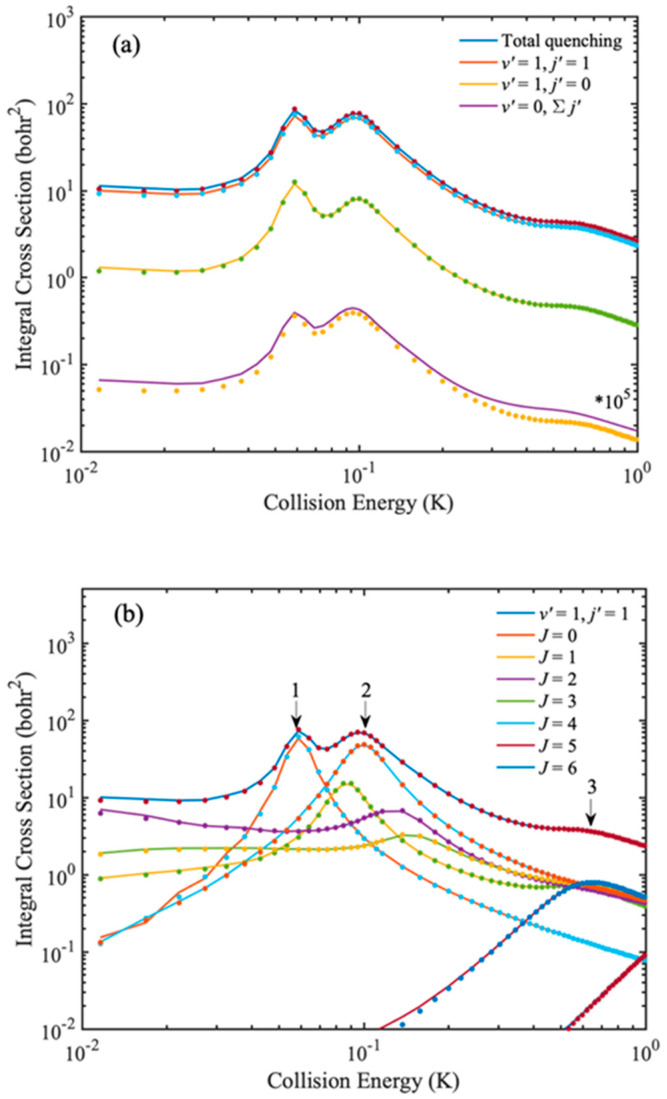
(**a**) State-resolved quenching ICSs of Na(3s) + HD(*v* = 1, *j* = 2) → Na(3s) + HD(*v′*, *j′*) collision. (**b**) *J* resolved quenching ICSs of Na(3s) + HD(*v* = 1, *j* = 2) → Na(3s) + HD(*v′* = 1, *j′* = 1) collision. The solid and dot lines are obtained by IALR-WP method and TICC method, respectively.

First, in order to verify that the IALR-WP method can accurately describe the dynamics of inelastic collisions in adiabatic triatomic systems, the ro-vibrational quenching ICSs of Na(3s) + HD(*v* = 1, *j* = 2) collision are calculated at low temperatures and compared with the accurate TICC results in Figure 2. The PES constructed by Hack et al. [35] is used in the calculations. The calculations are performed for *J* = 0~6 partial waves, and ro-vibrational quenching ICSs are obtained in collision energy range of 0.01 to 1 K. The numerical parameters used in the IALR-WP calculation are listed in Table 1. In the TICC calculations, energy cutoff is 3.0 eV, a total of 10 vibrational basis is used for *r* direction, and the other parameters are also carefully tested. As seen from Figure 2, the IALR-WP calculated ICSs are in good agreement with TICC results, which demonstrates the accuracy of IALR-WP method for characterizing adiabatic atom–diatom inelastic scatterings. The product ro-vibrational state resolved ICSs show that rotational quenching ICSs are much larger that of vibrational quenching. The total vibrational quenching ICS in Figure 2a is multiplied by 10 [5]. Three resonance peaks are found in the ICS curves. To obtain more insights into the formation of these peaks, the partial wave resolved inelastic scattering ICSs of Na(3s) + HD(*v* = 1, *j* = 2) → Na(3s) + HD(*v′* = 1, *j′* = 1) are shown in Figure 2b. As seen from Figure 2b, the first peak locates at 0.05 K, and mainly contributed by *J* = 0 partial wave. The second peak locates at 0.1 K, and *J* = 1, 2, 3 and 4 partial waves all contribute, but the contribution of *J* = 2 is the most obvious. The third peak locates at 0.6 K and it mainly contributed by *J* = 5 partial wave.

Next, we verify the accuracy of the IALR-WP method for inelastic scattering in non-adiabatic atom–diatom collisions at low temperatures. When the collision contains more than one electronic state, the diabatic representation is used in the WP calculation to avoid the singularity caused by the non-adiabatic couplings [36,37,38]. When the non-adiabatic coupling vanishes at long-range region, the IALR-WP method could be readily extended to non-adiabatic calculations as described in our previous work [32]. In such circumstances, the diabatic PESs approach to adiabatic PESs as *R* increases, therefore the initial wave packet could be constructed in diabatic representation. Additionally, the elastic and inelastic scattered wave packets can also be separated in diabatic representation by using IALR grid method. By using this strategy, the non-adiabatic IALR-WP calculations are performed for Na(3p) + H_2_(*v* = 0, 1) collisions in this work.

**Figure 3:**
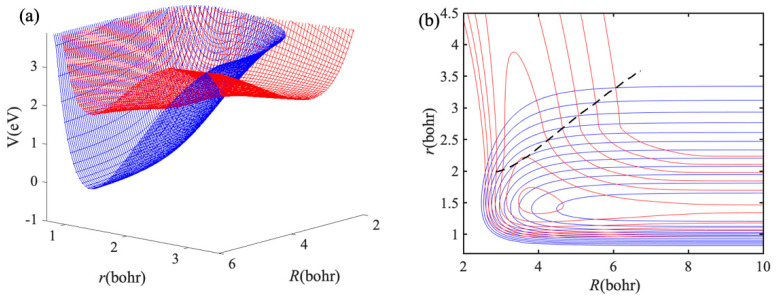
(**a**) A 3D plot of two lowest diabatic PESs for NaH_2_ at *C*_2*v*_ geometry. (**b**) Contour plot of (**a**), and the dash line represents the crossing of two diabatic PESs.

For the Na(3p) + H_2_ collision, the processes of electronic to vibrational and rotational energy transfer (Na(3p) + H_2_→ Na(3s) + H_2_ (*v′*, *j′*)) and chemical reaction (Na(3p) + H_2_→ NaH + H) through an excited state complex have been widely studied by experimentally and theoretically [39,40,41,42,43,44]. The electronic state of collision system changes during the collision for both electronic quenching and reactive collisions. To study the non-adiabatic collisions between Na(3p) and H_2_, Hack et al. [35] reported an analytical diabatic potential energy matrix for the two lowest electronic states of NaH_2_ system. The long-range forces between Na(3p) and H_2_ are included and non-diagonal matrix elements at long-range region are damped to zero in this non-adiabatic PESs, therefore it is a good candidate for testing the accuracy and ability of IALR-WP method for cold non-adiabatic collisions. For the Na(3p) + H_2_→ NaH + H reaction, it is an endothermic reaction and the endothermicity is about 0.47 eV, therefore the reaction channel is not considered in the current calculations. To better describe the non-adiabatic processes in the collision between Na(3p) and H_2_, a 3D plot of diabatic PESs as functions of *R* and *r* at C_2v_ geometry is shown in Figure 3a, and contour plot is shown in Figure 3b. There is an exciplex near the *R* = 4.2 *a*_0_ and *r* = 1.4 *a*_0_, and the *r* of the exciplex is close to the equilibrium bond length of H_2_ molecule. The black dash line in Figure 3b represents the crossing of two diabatic PESs. The minimum potential energy of the crossing line is almost close to the Na(3p) + H_2_ asymptotic energy, indicating that the electronic quenching should be no barriers. The values of *r* on the crossing line are greater than that equilibrium bond length of H_2_ molecule, thus H_2_ should be stretched before electronic quenching occurs. Therefore, a relatively large *r* grid range is required in the dynamics calculations of the Na(3p) + H_2_ collision.

**Figure 4:**
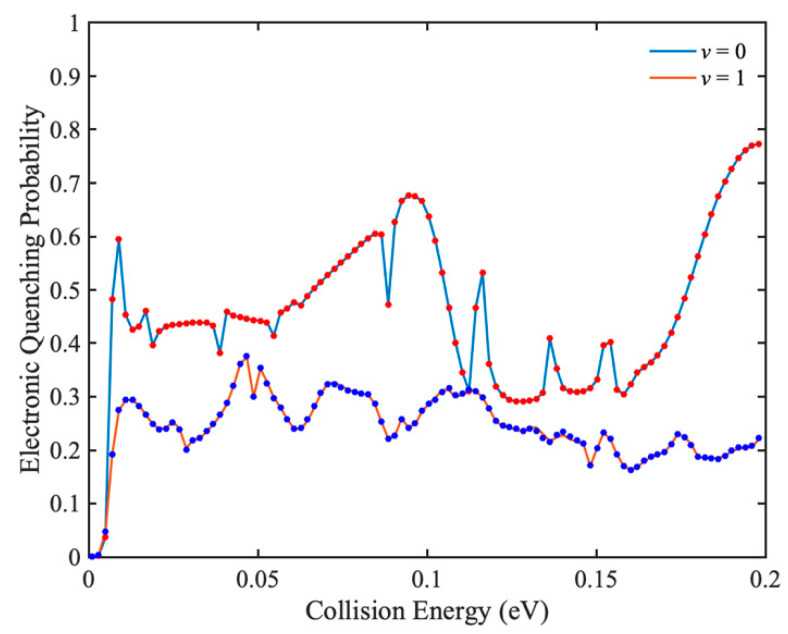
Total electronic quenching probabilities of Na(3p) + H_2_(*v* = 0, 1) → Na(3s) + H_2_ collisions, and solid and dot lines are obtained by IALR-WP method and L-shaped method, respectively.

To test the accuracy of IARL-WP method in characterizing electronic quenching scattering, the total electronic quenching probabilities of Na(3p) + H_2_(*v* = 0, 1) collisions are calculated at higher collision energies by IARL-WP method, and the results are compared with those of L-shaped WP method. In Figure 4, *J* = 0 total electronic quenching probabilities of Na(3p) + H_2_(*v* = 0, 1) collisions obtained by IALR-WP method and L-shaped WP method are compared. The agreement between two methods demonstrates the accuracy of the IALR-WP method in characterizing electronic quenching scatterings. There are many resonance peaks in the probability curves, which should be caused by the potential well on the excited state PES. In addition, although the vibrational excitation of H_2_ molecule opens up more product channels, it reduces the total electronic quenching probability. This is because there are ro-vibrational quenching channels Na(3p) + H_2_(*v′* = 0, *j′*) in addition to the electronic quenching channel in the Na(3p) + H_2_(*v* = 1) collision.

**Figure 5:**
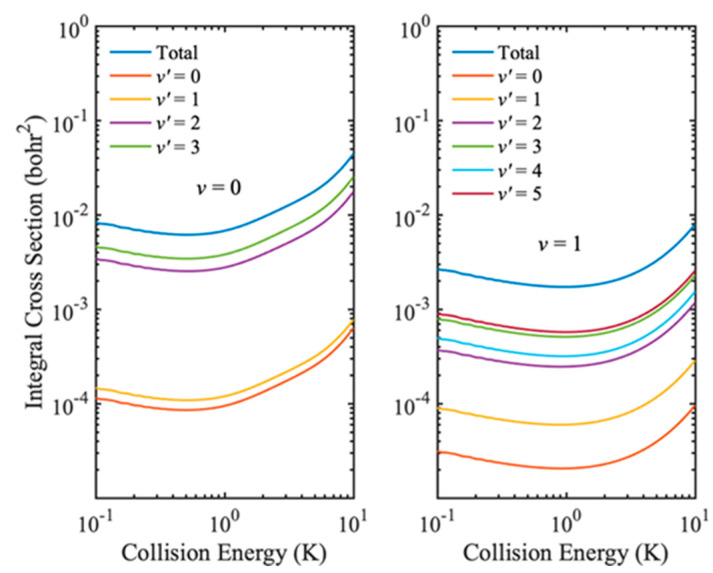
Electronic quenching probabilities of Na(3p) + H_2_(*v* = 0, 1) → Na(3s) + H_2_(*v′*) collisions.

**Figure 6:**
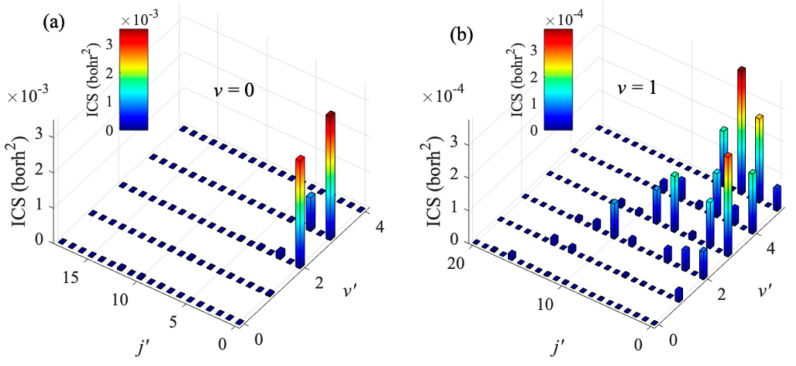
Ro-vibrational state distribution of H_2_ from electronic quenching of Na(3p) + H_2_(*v*) → Na(3s) + H_2_(*v′*, *j′*) collision with (**a**) *v* = 0, and (**b**) *v* = 1 at collision energy of 0.1 K.

After validating the IALR-WP method in treating non-adiabatic inelastic scatterings, it is employed to compute electronic quenching ICSs of Na(3p) + H_2_(*v* = 0, 1) collisions at low temperatures. Only *J* = 0~8 partial waves contribute to electronic quenching scattering in the collision energy range of 0.1 to 10 K. Since the electronic quenching of Na(3p) + H_2_ collision can produce molecules with very high ro-vibrational states, a relatively large amount of ro-vibrational basis is used in both the interaction region and the asymptotic region. The main parameters used in the IALR-WP calculations are listed in Table 2. In Figure 5, the electronic quenching ICSs of Na(3p) + H_2_ collision with two different initial vibrational states *v* = 0 and *v* = 1 are shown. It can be seen that the vibrational excitation of H_2_ molecule reduces the probability of electronic quenching, and the same result is obtained when the collision energy is high. In addition, the vibrational excitation of H_2_ molecule also has a great influence on the product state distribution. The products of Na(3p) + H_2_(*v* = 0) collision are mainly distributed in the *v′* = 3 state, while the products of Na(3p) + H_2_(*v* = 1) collision are mainly distributed in the *v′* = 5 state. To obtain more insights into the product state distribution, the ro-vibrational state distribution of Na(3p) + H_2_(*v* = 0, 1) collisions at collision energy of 0.1 K are shown in Figure 6. For the collision with *v* = 0, the products are mainly distributed in the rotational ground state and the vibrational excited state, which accords with the characteristics of the diabatic potential energy surface discussed above, and also indicates that the collision tends to occur in the T-geometry. When H_2_ molecule is excited to *v* = 1 state, the electronic quenching products have a wide rotational state distribution, but mainly distribute at lower rotational states.

**Figure 7:**
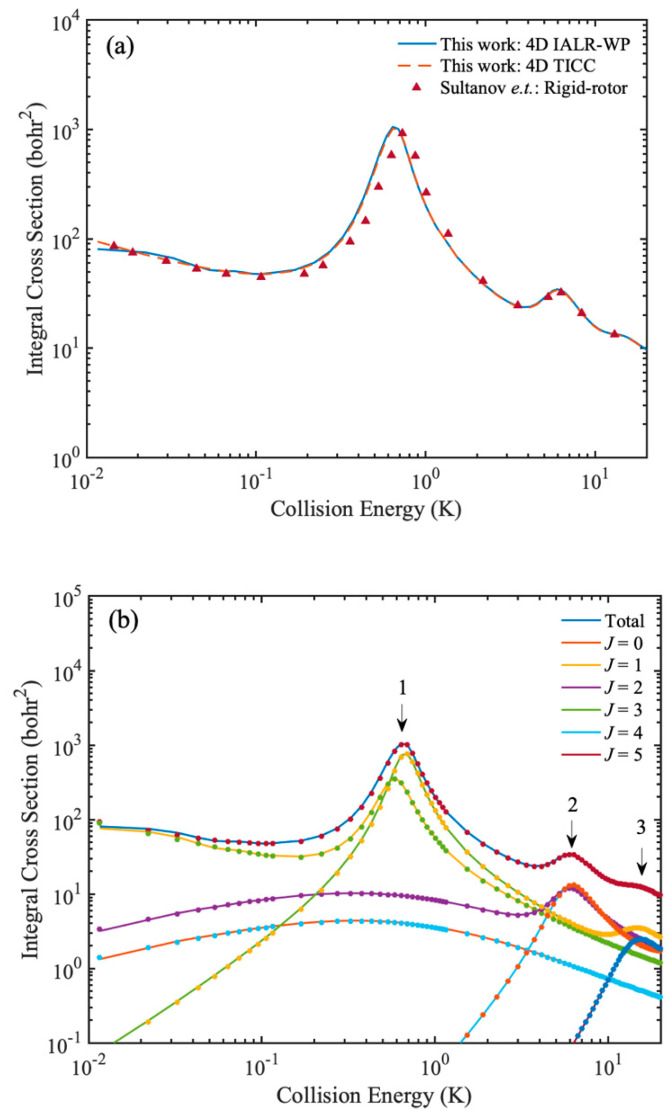
(**a**) Rotational quenching ICSs of HD(*v* = 0, *j* = 1) + H_2_(*v* = 0, *j* = 0) → HD(*v* = 0, *j* = 0) + H_2_(*v* = 0, *j* = 0) collision calculated by 4D TICC method and 4D IALR-WP method, and compared with the results calculated by Sultanov et al. by using rigid-rotor model. (**b**) *J* resolved rotational quenching ICSs of HD(*v* = 0, *j* = 1) + H_2_(*v* = 0, *j* = 0) → HD(*v* = 0, *j* = 0) + H_2_(*v* = 0, *j* = 0) collision. The solid and dot lines are obtained by IALR-WP method and TICC method, respectively.

Finally, we extend the IALR-WP method to diatom–diatom inelastic scattering at low temperatures. Since the H_2_ + H_2_ is the simplest neutral diatom–diatom system, the H_2_ + H_2_ and its isotopic variants are treated as a benchmark system to studying the quantum dynamics of diatom–diatom scatterings and testing new numerical calculation methods [45,46,47,48,49]. Here, we use the HD(*v* = 0, *j* = 1) + H_2_(*v* = 0, *j* = 0) → HD(*v* = 0, *j* = 0) + H_2_(*v* = 0, *j* = 0) collision as an example to illustrate the accuracy and ability of IALR-WP method in characterizing cold diatom–diatom inelastic scattering. The strategy is basically the same as in the case of atom–diatom scattering, except that the ro-vibrational quantum states of molecule are slightly more complicated for diatom–diatom collisions. For the HD(*v* = 0, *j* = 1) + H_2_(*v* = 0, *j* = 0) collision, Sultanov et al. [50] reported the rotational quenching ICS at low temperatures by using rigid-rotor model. To make a comparison with previous results, four-dimensional (one vibrational basis is used for *r*_1_ and *r*_2_) IALR-WP and TICC calculations are performed for HD(*v* = 0, *j* = 1) + H_2_(*v* = 0, *j* = 0) collision, and BMKP [51] PES is used. Main parameter used in the IALR-WP calculation are listed in Table 3. In the TICC method, *E_cut_* is set to 1.0 eV and the other parameters are chosen to ensure the convergence. The partial wave *J* is calculated from 0 to 5 and the converged ICSs are obtained at collision energy ranges from 0.01 to 20 K. A comparison of rotational quenching ICSs of HD(*v* = 0, *j* = 1) + H_2_(*v* = 0, *j* = 0) collision calculated by 4D TICC method, 4D IALR-WP method and rigid-rotor TICC method are shown in Figure 7a. The agreement of ICSs in Figure 7a demonstrates the accuracy of the results of 4D TICC and 4D IALR-WP in this work. To obtain more insights in to the contribution of partial waves for rotational quenching ICS, the partial wave resolved inelastic scattering ICSs are shown in Figure 7b. There are three peaks found in the total ICS of HD(*v* = 0, *j* = 1) + H_2_(*v* = 0, *j* = 0) → HD(*v* = 0, *j* = 0) + H_2_(*v* = 0, *j* = 0) collision. The first peak is located at 0.6 K, and mainly contributed by *J* = 1, 3 partial waves. The second peak is located at 6 K, and mainly contributed by *J* = 2, 4 partial waves. The third peak is located at 10.5 K and it mainly contributed by *J* = 3, 5 partial waves. The agreement between IALR-WP method and TICC method in partial wave resolved ICSs further demonstrate the accuracy of IALR-WP method and its ability in characterizing cold diatom–diatom inelastic scatterings.

**Figure 8:**
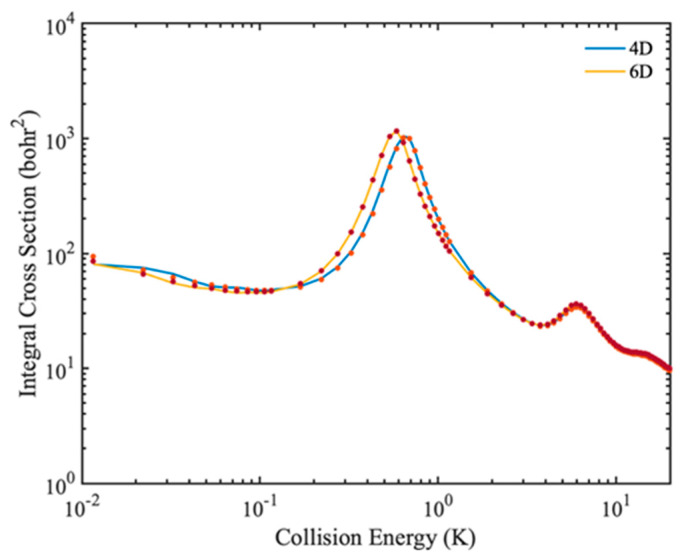
Comparison of rotational quenching ICSs of HD(*v* = 0, *j* = 1) + H_2_(*v* = 0, *j* = 0) → HD(*v* = 0, *j* = 0) + H_2_(*v* = 0, *j* = 0) collision calculated by 4D and 6D models. The solid and dot lines are obtained by IALR-WP method and TICC method, respectively.

In some cases, the rigid rotor approximation produces large errors, so full-dimensional quantum calculations are required to describe molecular collisions. In Figure 8, the 4D and 6D calculated rotational quenching ICSs of the HD(*v* = 0, *j* = 1) + H_2_(*v* = 0, *j* = 0) → HD(*v* = 0, *j* = 0) + H_2_(*v* = 0, *j* = 0) collision are shown. In the 6D calculation, four vibrational basis functions and three vibrational functions are used for HD and H_2_ molecule, respectively. Only small differences are found in the different dimensional calculated ICSs, indicating that the rotational quenching process of HD(*v* = 0, *j* = 1) + H_2_(*v* = 0, *j* = 0) → HD(*v* = 0, *j* = 0) + H_2_(*v* = 0, *j* = 0) can be described well by the rigid-rotor approximation. In addition, the results obtained by full dimensional IALR-WP method are in good agreement with those calculated by TICC method.

## 4. Conclusions

The quantum dynamics of molecular collisions at low temperatures are always characterized by the TICC method. Since the TICC method is difficult to extend to complex collision systems due to its steep numerical scaling law, the quantum dynamics calculations of full dimensional molecular collisions have been limited to three or four atom systems. The recent studies show that the IALR-WP method has great potential in the study of quantum dynamics of complex systems at cold and ultracold regimes. In this work, the IALR-WP method is extended to study the molecular inelastic scattering at low temperatures, and the accuracy of the method is verified in three different inelastic scattering cases.

Firstly, to demonstrate the accuracy of IALR-WP method in characterizing the adiabatic atom–diatom inelastic scattering, the ro-vibrational quenching ICSs of Na(3s) + HD(*v* = 1, *j* = 2) collision at cold temperatures is calculated, and compared with the results of TICC method. The calculation results show that the vibrational quenching ICS is much lower than that of rotational quenching ICS, and HD molecule is mainly distributed in the *v′* = 1, *j′* = 1 state. In addition, three resonance peaks are found in the ro-vibrational quenching ICSs, and the contribution of *J* partial waves to the resonances are analyzed.

Secondly, to demonstrate the accuracy of IALR-WP method in characterizing the non-adiabatic atom–diatom inelastic scatterings, the *J* = 0 electronic quenching probabilities of Na(3p) + H_2_(*v* = 0, 1) collisions are calculated by IALR-WP method, and the accuracy of the method is verified by comparison with the results of L-shaped WP method. The electronic quenching ICSs at low temperatures show that the vibrational excitation of H_2_ reduces the electronic quenching probability and has a great influence on the product state distribution.

Finally, the IARL-WP method is applied to HD(*v* = 0, *j* = 1) + H_2_(*v* = 0, *j* = 0) → HD(*v* = 0, *j* = 0) + H_2_(*v* = 0, *j* = 0) inelastic scattering to verify the accuracy of the IALR-WP method in characterizing diatom–diatom inelastic scatterings at low temperatures. Three resonance peaks are found on the rotational quenching ICS and the contribution of different *J* partial waves to those resonances are analyzed. In addition, only small differences are found in the 4D and 6D calculated ICSs, indicating that the HD(*v* = 0, *j* = 1) + H_2_(*v* = 0, *j* = 0) collision can be described well by the rigid-rotor approximation.

In recent years, with the development of experimental techniques, collision of highly excited state molecules has attracted extensive attention. Excited molecules, including rotational, vibrational and electronic excitation, can be prepared using several pumping techniques, even under ultracold conditions. The collision of excited state molecules, especially electronic excited state molecules, will produce product molecules with very high ro-vibrational states, which makes the TICC method very difficult. However, the IALR-WP method proposed in this work can describe such collision process effectively. Due to the better computational scaling in the IALR-WP method, this method is expected to be of great use in studying inelastic scattering involving heavy atoms and multi-electronic states, such as inelastic scattering of KRb + Rb, RbCs + Cs at low temperatures.

## Figures and Tables

**Table 1:** Numerical parameters used in IALR-WP calculations for the Na(3s) + HD(*v* = 1, *j* = 2) → Na(3s) + HD(*v′, j′*) collision at cold temperatures.

Parameter	Value
*R*	*R* ∈ [1.0, 3000.0], *N_R_*^III^ = 29,999, *N_R_*^II^ = 799, *N_R_*^I^ = 49
*r*	*r* ∈ [0.4, 6.0], *v*_Int_ = 15, *v*_Asy_ = 10
Rotational basis	*j*_Int_ = 20, *j*_Asy_ = 20
Damping function for *R*	*R_a_*^II^ = 40.0, *R_b_*^II^ = 80.0, *C_R_*^II^ = 0.005
	*R_a_*^III^ = 200.0, *R_b_*^III^ = 3000.0, *C_R_*^III^ = 0.00001
Initial wave packet	*R_c_* = 120.0, *δ* = 5.0, *E_c_* = 0.0008 eV
Propagation time	*T_tot_* = 100,000,000, Δ*_t_* = 10
Projection plane	*R_Inelastic_* = 35.0

**Table 2:** Numerical parameters used in IALR-WP calculations for the Na(3p) + H_2_(*v* = 0, 1) → Na(3s) + H_2_ collisions at low temperatures.

Parameter	Value
*R*	*R* ∈ [0.1, 3000.0], *N_R_*^III^ = 29,999, *N_R_*^II^ = 899, *N_R_*^I^ = 69
*r*	*r* ∈ [0.4, 8.0], *v*_Int_ = 70, *v*_Asy_ = 10
Rotational basis	*j*_Int_ = 40, *j*_Asy_ = 40
Damping function for *R*	*R_a_*^II^ = 40.0, *R_b_*^II^ = 90.0, *C_R_*^II^ = 0.01
	*R_a_*^III^ = 200.0, *R_b_*^III^ = 3000.0, *C_R_*^III^ = 0.00001
Initial wave packet	*R_c_* = 120.0, *δ* = 5.0, *E_c_* = 0.0008 eV
Propagation time	*T_tot_* = 100,000,000, Δ*_t_* = 10
Projection plane	*R_Inelastic_* = 35.0

**Table 3:** Numerical parameters used in IALR-WP calculations for the HD(*v* = 0, *j* = 1) + H_2_(*v* = 0, *j* = 0) → HD(*v* = 0, *j* = 0) + H_2_(*v* = 0, *j* = 0) collision at low temperatures.

Parameter	Value
*R*	*R* ∈ [2.0, 3000.0], *N_R_*^III^ = 29,999, *N_R_*^II^ = 799, *N_R_*^I^ = 69
Rotational basis for HD	*j*_Int_ = 4, *j*_Asy_ = 4
Rotational basis for H_2_	*j*_Int_ = 4, *j*_Asy_ = 4
Damping function for *R*	*R_a_*^II^ = 40.0, *R_b_*^II^ = 80.0, *C_R_*^II^ = 0.005
	*R_a_*^III^ = 200.0, *R_b_*^III^ = 3000.0, *C_R_*^III^ = 0.00001
Initial wave packet	*R_c_* = 120.0, *δ* = 8.0, *E_c_* = 0.0008 eV
Propagation time	*T_tot_* = 100,000,000, Δ*_t_* = 10
Projection plane	*R_Inelastic_* = 35.0

## Data Availability

The data that support the findings of this study are available from the corresponding author upon reasonable request.
